# Muay Thai exercises improve quality of life, love of life and self-control

**DOI:** 10.3389/fpsyg.2025.1584160

**Published:** 2025-05-23

**Authors:** Oktay Şahin, Coşkun Yılmaz, Süreyya Yonca Sezer, Fatma Neşe Şahin, Levent Ceylan, Baha Engin Çelikel, Çetin Tan, Mine Akkuş Uçar, Nagihan Kirikoğlu

**Affiliations:** ^1^Faculty of Sport Sciences, Gümüşhane University, Gümüşhane, Türkiye; ^2^Faculty of Sport Sciences, Munzur University, Tunceli, Türkiye; ^3^Faculty of Sport Sciences, Ankara University, Ankara, Türkiye; ^4^Faculty of Sport Sciences, Hitit University, Çorum, Türkiye; ^5^Faculty of Sport Sciences, Firat University, Elazığ, Türkiye; ^6^Faculty of Sport Sciences, Mardin Artuklu University, Mardin, Türkiye; ^7^Faculty of Sport Sciences, Gazi University, Ankara, Türkiye

**Keywords:** combat sports, mental health, Muay Thai, love of life, self-control, quality of life, sport psychology

## Abstract

**Background:**

The existing research on Muay Thai sports has focused predominantly on the physiological effects of training, with limited attention devoted to the study of quality of life, love of life and self-control. The present study examined the effects of Muay Thai exercises on quality of life, love of life and self-control scores in healthy male subjects.

**Methods:**

The present study comprised 50 healthy sedentary male subjects. The subjects were randomly divided into two groups: one group engaged in Muay Thai training (MTT), while the other group served as a control group (CON). The sample sizes for the MTT and CON groups were both 25. The MTT group participated in basic Muay Thai training, while the CON group continued their normal life regime. The SF-12 quality of life scale, love of life scale and multidimensional self-control scale were administered before and after the six-week training period.

**Results:**

The study concluded that the six-week Muay Thai training program had a significant effect on quality of life levels, with 13.23% (*p* = 0.003) and 21.93% (*p* < 0.001) of participants demonstrating improvements in physical and mental scores, respectively. In terms of self-control levels, the program was found to have a significant effect on initiation and inhibition scores, with increases of 23.78% (*p* = 0.001) and 24.69% (*p* < 0.001), respectively. It was concluded that had a significant effect on the sub-dimensions of the Love of Life scale with increases of Positive Attitude Toward Life (PAWL) 18.63% (*p* < 0.001), Happy Results of the Love of Life (HRLL) 20.11% (*p* < 0.001) and Meaningfulness of Life (ML) 15.62% (*p* < 0.001), respectively. However, no significant differences were detected in any of the scales within the control group.

**Conclusion:**

Muay Thai exercises had a positive effect on quality of life, love of life and self-control levels in healthy male subjects. By providing valuable insights into how Muay Thai exercise affects quality of life, love of life, and self-control, this research can guide future intervention and program design in the context of sport psychology.

## 1 Introduction

In today’s world, regular physical activity plays a vital role in helping individuals maintain both physical and psychological well-being (Herbert, 2022; Mahindru et al., 2023). Research has shown that regular physical activity supports physiological balance and has positive effects in various areas such as mobility, functionality, quality of life, and overall life satisfaction (Faustino and Neves, 2020; Kliziene et al., 2021). Additionally, it has been noted that physical activity promotes the release of hormones associated with happiness and relaxation, serving as a source of motivation and a preventive tool for individuals (Brito et al., 2020; Martín-Rodríguez et al., 2024; Wang et al., 2025a).

In this context, martial arts offer a holistic approach that goes beyond being merely a form of physical exercise, supporting the psychosocial development of individuals (Pedrini and Jennings, 2021). In particular, Muay Thai, a martial art originating from Taiwan, plays a significant role not only in developing physical skills but also in enhancing multiple dimensions that influence an individual’s quality of life (Prasetyo et al., 2024; Wang et al., 2025b). Over time, Muay Thai has transformed into a global phenomenon, gaining popularity across different age groups and social segments, and has become a notable domain for both personal development and social participation (Vail, 2014; Soontayatron, 2025).

There is a growing body of literature examining the effects of Muay Thai. For instance, Pessina (2017) emphasized that Muay Thai contributes significantly to physical competence, self-discipline, and self-confidence. Similarly, de Oliveira et al. (2023) highlighted the sport’s positive impact on psychological well-being and overall quality of life. Moreover, researchers such as Utesch et al. (2018) have shown that martial arts strengthen individuals in psychosocial aspects such as social belonging, self-expression, and stress management.

In the domain of combat sports, Muay Thai has been identified as a notable modality due to its intermittent or interval nature (Crisafulli et al., 2009). The utilization of Muay Thai for the purposes of promoting the physiological development of children has been documented (Saraiva et al., 2024a; Saraiva et al., 2024b; Saraiva et al., 2023; Saraiva et al., 2022; Saraiva et al., 2018). Furthermore, the efficacy of Muay Thai in facilitating the physical development of healthy women has been demonstrated (Rapkiewicz et al., 2018), injuries sustained by adult individuals (Gartland et al., 2001), technique and conditioning (Turner, 2009), physical development (de Oliveira et al., 2023), balance and posture control (Quintero et al., 2024), physical fitness, speed and strength (Souza and Farje, 2019), cardiovascular health (Elızıárıo et al., 2019) and cognitive performance (Hunt, 2005). Despite the plethora of studies investigating the psychological effects of other martial arts, the paucity of research on psychosocial aspects in Muay Thai is striking (Saraiva et al., 2024a; Muller-Junior and Capraro, 2024; de Oliveira et al., 2023).

Studies on mental health factors such as quality of life, love of life, and self-control are often cross-sectional in design, indicating the need for longitudinal interventions (Ong and Ruzmin, 2015; Davies and Deckert, 2020; Croom, 2022; de Oliveira et al., 2023). The aim of the present study was to analyze the effects of a six-week Muay Thai program on quality of life, love of life, and self-control levels among university students. The main hypothesis of the study is that 6-week MT exercises will improve quality of life, love of life and self-control levels in healthy male individuals.

## 2 Materials and methods

### 2.1 Participants

The study included 50 healthy sedentary male subjects. GPower 3.1 program was used to determine the required number of participants. The results of the power analysis sampling study showed that the study could be completed with 20 subjects in each group (effect size: 0.80; actual power: 0.89). To account for potential losses, an additional 25% was added for a total of 50 subjects, with 25 subjects allocated to each group (Control and Muay Thai). In order to determine in which group the subjects forming the sample would be included, numbers between 1 and 50 were randomly assigned to two groups through a computerized program.^1^ Participants with chronic or any disease were excluded from the study. Verbal and written informed consent was obtained from all participants before starting the study. Participants included in the study were healthy individuals with no existing health problems and no history of illness, who were not using any medication based on their physical and mental health evaluations. Age, height, weight, and daily physical activity levels of the participants were assessed, and their previous involvement in sports was identified and recorded. A data collection form was created for this purpose.

### 2.2 Experimental design

**Figure 1:**
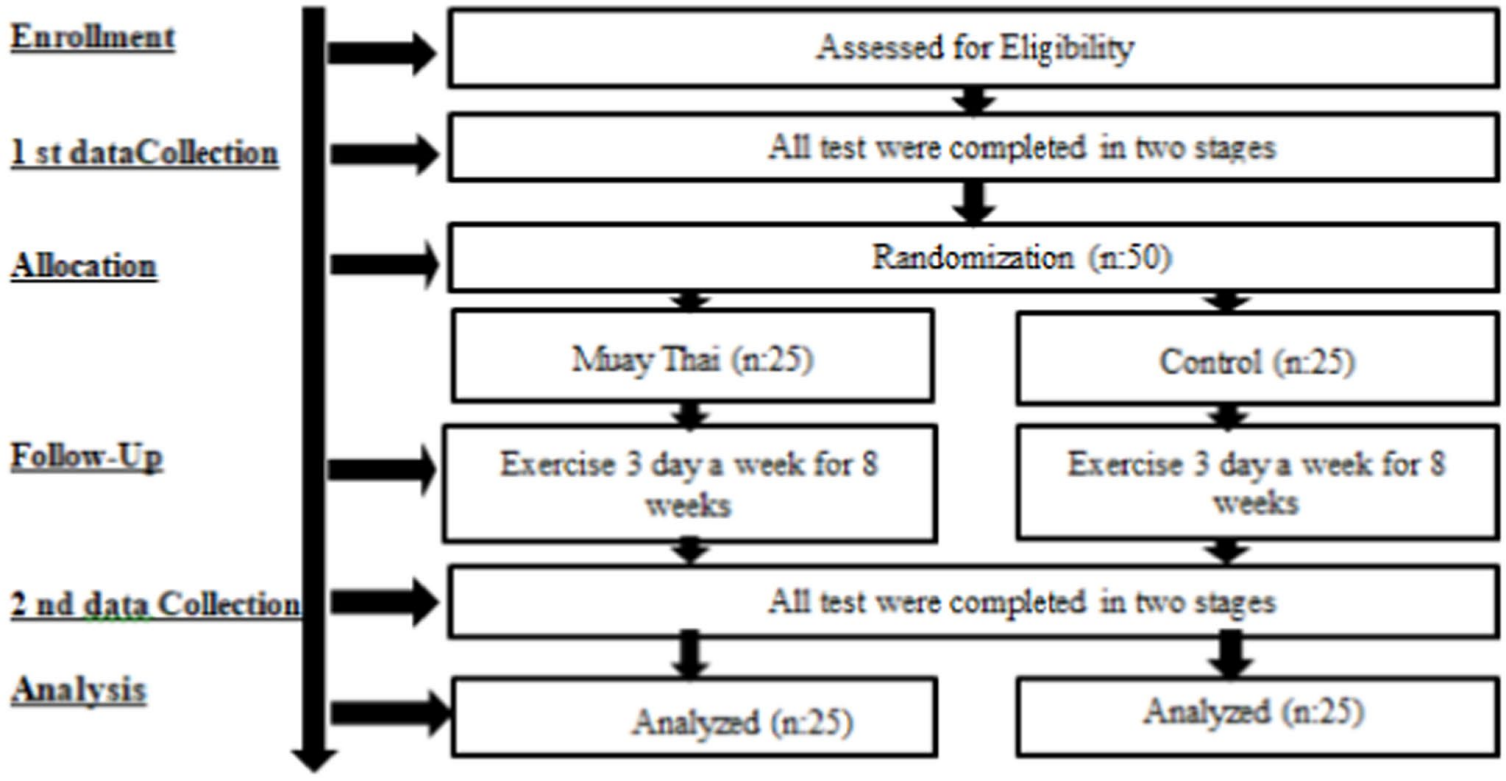
Experimental design.

The study was designed as a randomized, controlled experimental study. Participants were randomly assigned to two different groups: Muay Thai Training (MTT) group and Control group (CON). Prior to the commencement of the study, male participants were informed about the training procedures and scales. Each subject was given a detailed explanation about the Muay Thai training procedure. Subsequent to this preliminary information session, a period of 1 week elapsed prior to the commencement of the exercise training regime, during which time the subjects’ pre-exercise measurements were obtained and recorded. Subsequent to the culmination of the six-week training program, final measurements were obtained (Figure 1).

### 2.3 Body composition measurement

In the study, body composition was measured with Gaia 359 Plus Body-pass bioelectrical impedance analyzer to indicate that the participants had similar body characteristics. The Gaia 359 Plus BodyPass was used to determine the subject’s height and body weight (Table 1).

**Table 1:** Descriptives.

	Height		Weight		Age	
Group	X	S.D	X	S.D	X	S.D
Muay thai	174.04	9.15	69.42	12.67	22.16	1.23
Control	174.52	6.79	68.86	6.90	22.24	1.44

X; Mean, S.D; Standard deviation.

### 2.4 Love of life scale

The scale developed by Yıldırım and Özaslan (2022) measures a person’s general positive attitude toward life or enjoyment of life. The scale consists of three factors: positive attitude toward life (PAWL) (8 items), happy results of love of life (HRLL) (4 items) and meaningfulness of life (ML) (4 items). Responses range from 1 to 5 on a 5-point Likert-type scale (1 = no and 5 = very much). Sample items for each factor are ‘Life is full of pleasure’ for positive attitude toward life, ‘Love of life adds beauty’ and ‘I would like to have a long life to achieve what I hope for’ for happy outcomes of love of life. High scores indicate a positive attitude toward the meaningfulness of life, happiness and meaningfulness of life. Cronbach’s alpha coefficients were calculated as 0.84 for the positive attitude toward life sub-dimension and 0.81 for the happy results of love of life sub-dimension (Yıldırım and Özaslan, 2022).

### 2.5 Quality of life scale (QoL)

Short Form-36 (SF-36) was developed by Ware et al. (1995) to measure quality of life. SF-12 Quality of Life Scale was formed by taking 12 different items from 8 subheadings of SF-36. Turkish validity and reliability study was conducted by Soylu and Kütük (2021). In the scale, the functional status, well-being and general health perception of the individual are asked. Questions questioning physical and emotional status are answered as yes or no, and other questions include Likert-type options ranging between 3 and 6. Mental component summary (MCS) score is obtained from mental health, emotional role, social functioning and energy subcategories and physical component summary (PCS) score is obtained from physical role, physical functioning, general health and body pain subcategories. Scoring ranges from 0 to 100. A higher score is an indicator of better health.

The calculation of sub-dimensions in the obtained data was made on https://orthopowertools.com/SF12. The Cronbach’s alpha coefficient of the SF-12 Short Health Scale was calculated as *α*: 0.73 in the physical subscale and *α*: 0.72 in the mental subscale (Soylu and Kütük, 2021). In our study, it was calculated as *α*: 727 in the physical sub-dimension and *α*: 742 in the mental sub-dimension.

### 2.6 The multidimensional self-control scale (MSCS)

The scale developed by Nilsen et al. (2020) and subsequently adapted into Turkish by Koç et al. (2023) comprises eight items and two sub-dimensions, namely Initiation and Inhibition. The Initiation sub-dimension comprises 2, 4, 5 and 8 items, while the Inhibition sub-dimension consists of 1, 3, 6, and 7 items. In order to ensure consistency and comparability, items one, three, five and seven have been reverse scored. The scale is of the five-point Likert type. In the present study, the alpha coefficient was calculated as 0.661 for the initiation subscale and 0.611 for the inhibition subscale. It was determined that the scale has highly reliable results of 0.60 ≤ *α* < 0.80.

### 2.7 Weekly training program

Muay Thai training sessions were conducted over a period of 6 weeks, with the initial 4 weeks encompassing fundamental movements and techniques. The subsequent 2 weeks were designed to familiarize participants with combined strikes and techniques (Delp, 2005). The Muay Thai training program was overseen by a third-level expert coach.

Training sessions were conducted thrice weekly for a duration of one and a half hours, on non-consecutive days. Protective equipment such as gloves, Thai pads, armbands, and head and rib protectors was utilized. The training intensity was maintained within the moderate to high range on the basis of the perceived exertion. Perceived exertion was measured using the RPE scale. The Rate of Perceived Exertion (RPE) represents how hard you feel your body is working during physical activity. It is subjective, meaning it is based on how hard you personally feel you are exerting yourself during exercise (Borg et al., 1987) (Table 2).

**Table 2:** Weekly training program.

Week	Applied movements
1 Week	Muay Thai guard stance, Punch, Matt Naa Dhrong, Maat Suey, Matt Lang Dhrong, Djab-Ko (Clinch) (Each set of movements was repeated 3 times for 4 min. Rest was given for 1 min between each set.)
2 Week	Elbow, Sok Tee, Sawk At, Sawk Tad, Sawk Wiang (Each set of movements was repeated 3 times for 4 min. Rest was given for 1 min between each set.)
3 Week	Kick, Tae Kod, Tae Glaang, Tae Tud, Teep Dhrong / Teep Dhob (Each set of movements was repeated 3 times for 4 min. Rest was given for 1 min between each set.)
4 Week	Knee, Kao Youwn / Kao Dhrong, Ka Wiang, Kao Dhob / Kao Chiang (Each set of movements was repeated 3 times for 4 min. Rest was given for 1 min between each set.)
5 Week	Combined stroke training (Each set of combined movements was repeated 3 times for 4 min. 1 min rest was given between each set).
6 Week	Combined stroke training (Each set of combined movements was repeated 3 times for 4 min. 1 min rest was given between each set).

### 2.8 Statistical analysis

Statistical analyses were performed via SPSS (Version 21.0 for Windows, Chicago, IL, USA) software, with the statistical significance set at 0.05. The Shapiro–Wilk normality test was performed to determine the homogeneity of the sample. Each pre-test and post-test differences were determined by paired comparison test (paired *t*-test), and inter-group differences were determined by one-way analysis of variance with post-test and pre-test difference values. In addition, in the comparison of paired groups, the effect size was calculated according to Cohen’s *d* formula [(M2 - M1) / SD pooled]. Moreover, it was interpreted as follows: 0–0.19 insignificant, 0.20–0.59 small, 0.6–1.19 moderate, 1.20–1.99 large, and ≥2.00 very large (Cohen, 2013) (Table 3).

**Table 3:** Mean, reliability, skewness and kurtosis values of the scales.

(n:50)	Sub-dimension	Number of items	X	S.D	α	p.	Kurtosis	Skewness
Quality of life scale (QOLS) (*α*: 0.715)	Physical	4	49.69	6.32	0.727	0.004	−1.028	1.455
	Mental	8	44.44	9.46	0.742	0.028	−0.665	0.304
The multidimensional self-control scale, MSCS (*α*: 0.686)	Inhibition	4	13.83	3.06	0.574	0.049	−0.082	−0.678
	Initiation	4	14.03	3.01	0.632	0.007	−0.100	−0.419
Love of life scale (*α*: 0.956)	PAWL	8	27.22	7.21	0.925	0.162	−0.254	−0.546
	HRLL	4	13.71	3.50	0.837	0.010	−0.118	−0.430
	ML	4	14.30	3.57	0.802	<0.001	−0.297	−0.658

X; Mean, S.D; Standard deviation, *α*; Cohen’s d effect size, *p*; Significance level.

## 3 Results

**Figure 2:**
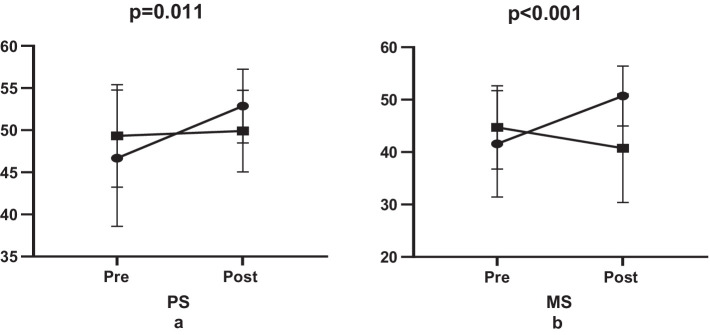
Comparison of quality of life levels.

The study concluded that a six-week Muay Thai training regime had a significant impact on both physical and mental scores, with a respective effect of 13.23% (e.s = 0.95, *p* = 0.003, Figure 2a) and 21.93% (e.s = 1.11, *p* < 0.001, Figure 2b). However, in the control group, while there was no effect on the physical score (1.17%, e.s = 0.11, *p* = 0.580, Figure 2a), a significant negative effect was observed on the mental score either, despite a decrease of −8.83% (e.s = 0.43, *p* = 0.044) (Figure 2b).

**Figure 3:**
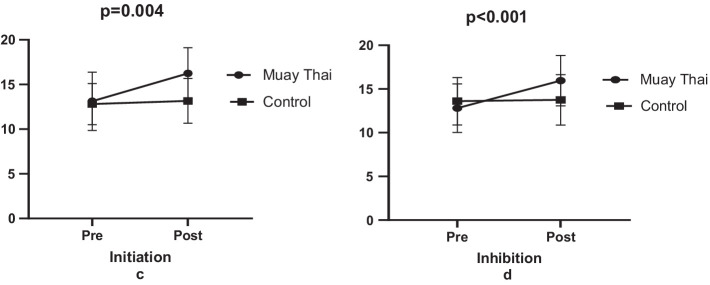
Comparison of self-control levels.

The study concluded that the six-week Muay Thai training regime had a significant effect on both initiation, and inhibition scores, with effect of 23.78% (e.s = 1.02, *p* = 0.001, Figure 3c) and 24.69% (e.s = 1.12, *p* < 0.001, Figure 3d), respectively. However, in the control group, no significant differences were observed in initiation scores (2.81%, e.s = 0.15, *p* = 0.195, Figure 3c) or inhibition scores (1.17%, e.s = 0.06, *p* = 0.491, Figure 3d) (Figure 3).

**Figure 4:**
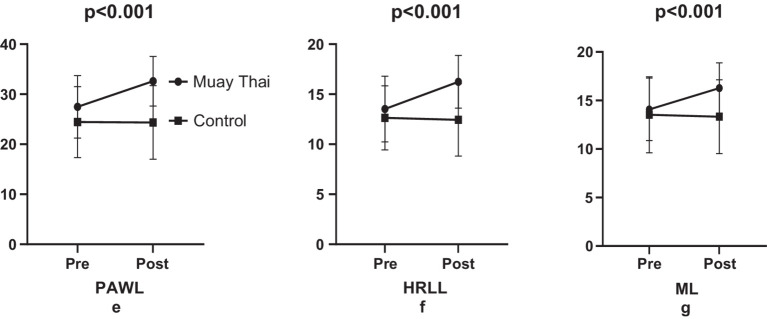
Comparison of love of life levels.

The study concluded that the six-week Muay Thai training regime had a significant effect on the subscales of the Love of Life scale, with increases of PAWL %18.63 (e.s = 0.91, *p* < 0.001, Figure 4e), HRLL %20.11 (e.s = 0.91, *p* < 0.001, Figure 4f) ve ML 15.62 (e.s = 0.75, *p* < 0.001, Figure 4g), respectively. However, in the control group, no significant difference was observed in PAWL by −0.32% (e.s = 0.01, *p* = 0.792, Figure 4e), HRLL by −1.58% (e.s = 0.06, *p* = 0.495, Figure 4f) and ML by −1.47% (e.s = 0.05, *p* = 0.618, Figure 4g) (Figure 4).

## 4 Discussion

The main findings of this study indicate that Muay Thai training significantly improved quality of life, love of life, and self-control levels. Following Muay Thai training, participants showed increases in quality of life scores, with a 13.23% improvement in physical scores and a 21.93% improvement in mental scores. In terms of self-control, there were increases of 23.78% in the initiation subscale and 24.69% in the inhibition subscale. Regarding love of life, the increases were 18.63% in PAWL, 20.11% in HRLL, and 15.62% in ML. In contrast, the control group exhibited only minor, statistically insignificant improvements. These findings suggest that the intervention applied to the Muay Thai exercise group effectively enhanced quality of life, love of life, and self-control levels as assessed in this study.

Muay Thai training has been demonstrated to have a positive impact on the well-being of adolescents, functioning as a preventative measure and a health promoter (Saraiva et al., 2024a). The practice has been shown to facilitate the release of hormones associated with feelings of happiness, relaxation and general well-being. Furthermore, a study conducted on women reported that Muay Thai exercises, when utilized as a therapeutic modality, have the capacity to alleviate symptoms and enhance quality of life in cases of depression, insomnia, muscle discomfort and even stressful situations (de Oliveira et al., 2023).

As Brito et al. (2020) demonstrate, Muay Thai training confers a number of benefits to the practitioner, including moral, physical and psychological advantages. From a moral standpoint, practitioners cultivate the capacity to embrace both triumph and defeat with equanimity, act with humility, show deference to their opponents, and maintain discipline not only in sports but also in other domains of life. The psychological benefits of exercise are well-documented, with the production of hormones associated with happiness and relaxation being a key benefit. Consequently, the practice of Muay Thai has been shown to reduce the effects of stress, enhance reasoning abilities, alleviate psychological tension, and boost self-confidence (Schneider, 2022). From a physiological standpoint, the most sought-after benefits among athletes include muscle mass gain, cardiovascular development, and the improvement of psychomotor skills and motor coordination. According to Graça and Sılva (2015), the practice of Muay Thai offers several advantages, including the enhancement of coordination and motor skills, an increase in social responsibility, and the promotion of overall health.

This article sets out the findings of research which indicates that Muay Thai training has a significant role to play in enhancing practitioners’ mental health and quality of life. Research has demonstrated that this discipline integrates combat techniques with rigorous physical training, providing a comprehensive approach to well-being (Saraiva et al., 2018; Saraiva et al., 2024b). The practice of physical activity is closely linked to the harmonious balance of physiological and psychosocial factors within the body (Li and Guo, 2023). When performed regularly, it plays a fundamental role in the prevention and management of chronic non-communicable diseases by promoting better mobility, functional capacity, and quality of life at all stages of life (Faustino and Neves, 2020).

In the study, a six-week Muay Thai training regime had a significant effect on physical (13.23%) and mental (21.93%) scores in quality of life levels. A study conducted on obese children demonstrated that Muay Thai could improve pain scores, a key component of quality of life (Saraiva et al., 2024a). Beyond its positive effects on physical and mental health, Muay Thai also serves as a strong protective factor against stress. It has been concluded that combat sports of this nature positively impact practitioners’ health and quality of life (da Luz Filho et al., 2024). In a study conducted on women, de Oliveira et al. (2023) reported that Muay Thai training had positive effects on anxiety control, social engagement, bodily changes, and various quality of life parameters, confirming the sport’s potential to enhance practitioners’ overall well-being. Similarly, a Muay Thai project led by Campos and Pontes (2015) demonstrated that the sport provides multiple benefits to practitioners’ quality of life. As individuals feel more competent and confident, their self-esteem and self-worth are also positively influenced. Therefore, Muay Thai is not only an effective form of self-defense but also a valuable tool for improving health and quality of life for those who incorporate it into their routines (Campos and Pontes, 2015).

The study found that the six-week Muay Thai martial arts training regime had a significant effect on the levels of initiation (23.78%) and inhibition (24.69%) in the self-control subdimension. dos Santos et al. (2021) emphasized the importance of Muay Thai practices in developing discipline and posture, increasing self-control, improving physical condition and supporting muscle mass gain. The study by Brito et al. (2020) revealed that women who practice Muay Thai reported positive contributions such as weight loss, body shaping, self-confidence, self-control, happiness and relaxation. The findings of Stepanyan and Nadoyan (2024) further substantiate the notion that Muay Thai training fosters a decline in aggressive behavior, alongside the cultivation of self-control and respect.

In the study, it was concluded that the six-week Muay Thai martial arts training regime had a significant effect on the sub-dimensions of the Love of Life scale, with increases of 18.63% in PAWL, 20.11% in HRLL and 15.62% in ML. To the best of our knowledge, the Love of Life Scale has been applied for the first time to individuals receiving Muay Thai combat sports training. We believe that these findings will contribute to the existing Muay Thai literature. Muay Thai is widely recognized as a combat sport that offers numerous benefits to its practitioners. However, it is well understood that effective training in this sport requires careful consideration of key parameters such as volume, intensity, duration, frequency, and complexity. Existing literature supports the notion that Muay Thai training provides benefits across different genders and age groups (Elızıárıo et al., 2019; de Sousa et al., 2017; Souza and Farje, 2019; Batista, 2019).

It is evident that no study has hitherto analyzed the effects of Muay Thai training on love of life, self-control and quality of life among university students. This lacuna can be regarded as a notable strength of the present study. Moreover, the utilization of Muay Thai, a widely practiced combat sport, in conjunction with the incorporation of random sampling methodologies, serves to further augment the study’s methodological rigor.

This study has several limitations. The small sample size restricted the ability to generalize the findings. The research focused on a six-week time frame and did not assess long-term outcomes, which limits the understanding of sustainable effects. All participants were from a single department at one university, which restricts the applicability of the results to other student populations. The study did not address potential gender differences that could influence the outcomes. Future research should aim to include larger and more diverse samples, examine long-term effects, consider gender as a variable, and explore a broader range of Muay Thai exercise protocols to provide a more comprehensive understanding.

## 5 Conclusion

This study provides compelling evidence that the integration of Muay Thai combat sports exercises into the academic curriculum of university students significantly improves various aspects of sport psychology and mental health. Our findings show that beginner-level Muay Thai combat sports exercises improve levels of love of life, self-control, and quality of life. These results underscore the critical role of Muay Thai combat sports exercises in the optimization of mental health and sport psychology. The observed improvements in love of life and quality of life, combined with increased levels of self-control, suggest that Muay Thai exercises make a valuable contribution to mental health and sport psychology.

## Data Availability

The original contributions presented in the study are included in the article/Supplementary material, further inquiries can be directed to the corresponding author.

## Footnotes

^1^ https://www.randomizer.org/

## References

[ref1] Batista M. H. (2019). Perfil antropométrico e das capacidades físicas de praticantes de Muay Thai do sexo feminino da cidade de Ouro Preto-MG. Ouro Preto: Universidade Federal de Ouro Preto.

[ref2] Borg G. Hassmen P. Lagerstrom M. (1987). Perceived exertion related to heart rate and blood lactate during arm and leg exercise. Eur. J. Appl. Physiol. Occup. Physiol. 56, 679–685. doi: 10.1007/BF00424810, PMID: 3678222

[ref3] Brito R. F. de Holanda Bastos P. A. Brasileiro F. C. (2020). A participação da mulher no muay thai. Braz. J. Dev. 6, 18095–18112. doi: 10.34117/bjdv6n4-106

[ref4] Campos W. M. Pontes J. A. M. (2015). Lutas em foco: o Muay Thai e a mudança de comportamento dos alunos da universidade federal do Ceará. FIEP Bull. 85, 539–543. doi: 10.16887/85.a1.92

[ref6] Cohen J. (2013). Statistical power analysis for the behavioral sciences. New York: Routledge.

[ref7] Crisafulli A. Vitelli S. Cappai I. Milia R. Tocco F. Melis F.. (2009). Physiological responses and energy cost during a simulation of a Muay Thai boxing match. Appl. Physiol. Nutr. Metab. 34, 143–150. doi: 10.1139/H09-002, PMID: 19370044

[ref8] Croom A. M. (2022). Muay Thai, psychological well-being, and cultivation of combat-relevant affordances. Philosophies 7:65. doi: 10.3390/philosophies7030065

[ref9] da Luz Filho M. A. Leite C. C. N. Pires M. R. Ribeiro V. B. Muay thaı e seus benefícıos a saúde de jovens e adultos. Seminário Nacional e Seminário Internacional Políticas Públicas, Gestão e Práxis Educacional. (2024); 6128–6143.

[ref10] Davies S. G. Deckert A. (2020). Muay Thai: women, fighting, femininity. Int. Rev. Sociol. Sport 55, 327–343. doi: 10.1177/1012690218801300

[ref11] de Oliveira B. H. S. de Araújo C. Q. L. Arantes L. M. (2023). Os benefícios da prática do Muay Thai na qualidade de vida: uma perspectiva feminina. Res. Soc. Dev. 12:e19212742753. doi: 10.33448/rsd-v12i7.42753

[ref12] de Sousa B. R. G. de Oliveira T. D. Sabino G. S. (2017). Aplicação da avaliação funcional de movimento (fms) em praticantes de muay thai de Belo Horizonte/MG. Rev. Interdiscip. Ciênc. Méd. 1, 51–61.

[ref13] Delp C. (2005). Muay Thai basics: Introductory Thai boxing techniques. California: Blue Snake Books.

[ref9001] dos Santos R. F. do Carmo Leite T. L. Lima B. N. de Santana Maneschy M. de Assis Junior R. S. da Silva Almeida K. (2021). Capacidades físicas na prática do muay thai. Revista CPAQV–Centro de Pesquisas Avançadas em Qualidade de Vida. 13:2. doi: 10.36692/v13n3-03R

[ref14] Elızıárıo D. D. Elizângela F. F. S. Lavorato V. N. Olıveıra R. A. R. (2019). Aptidão física em praticantes de muay thai do sexo feminino. Cadern. Cientí. Fagoc Grad. Pós-Grad. 6, 29–36.

[ref15] Faustino A. M. Neves R. (2020). Benefícios da prática de atividade física em pessoas idosas: revisão de literatura. Rev. Eletrônica Acervo Saúde 12:e3012. doi: 10.25248/reas.e3012.2020

[ref16] Gartland S. Malik M. H. A. Lovell M. E. (2001). Injury and injury rates in Muay Thai kick boxing. Br. J. Sports Med. 35, 308–313. doi: 10.1136/bjsm.35.5.308, PMID: 11579062 PMC1724381

[ref17] Graça R. L. Sılva A. V. (2015). Muay Thai: benefícios comportamentais nas crianças praticantes na cidade de Cocal do Sul –SC. Humanidades, ciências e educação -UNESC. Available online at: http://repositorio.unesc.net/tutam/1/3095 (Accessed May, 22, 2024).

[ref18] Herbert C. (2022). Enhancing mental health, well-being and active lifestyles of university students by means of physical activity and exercise research programs. Front. Public Health 10:849093. doi: 10.3389/fpubh.2022.849093, PMID: 35548074 PMC9082407

[ref19] Hunt L. (2005). Ong-Bak: new Thai cinema, Hong Kong and the cult of the ‘real’. New Cin. 3, 69–84. doi: 10.1386/ncin.3.2.69/1

[ref20] Kliziene I. Cizauskas G. Sipaviciene S. Aleksandraviciene R. Zaicenkoviene K. (2021). Effects of a physical education program on physical activity and emotional well-being among primary school children. Int. J. Environ. Res. Public Health 18:7536. doi: 10.3390/ijerph18147536, PMID: 34299987 PMC8304760

[ref21] Koç H. Şimşir Gökalp Z. Seki T. (2023). The relationships between self-control and distress among emerging adults: a serial mediating roles of fear of missing out and social media addiction. Emerg. Adulthood 11, 626–638. doi: 10.1177/21676968231151776

[ref22] Li Y. Guo K. (2023). Research on the relationship between physical activity, sleep quality, psychological resilience, and social adaptation among Chinese college students: a cross-sectional study. Front. Psychol. 14:1104897. doi: 10.3389/fpsyg.2023.1104897, PMID: 36844303 PMC9950505

[ref23] Mahindru A. Patil P. Agrawal V. (2023). Role of physical activity on mental health and well-being: a review. Cureus 15:e33475. doi: 10.7759/cureus.33475, PMID: 36756008 PMC9902068

[ref24] Martín-Rodríguez A. Gostian-Ropotin L. A. Beltrán-Velasco A. I. Belando-Pedreño N. Simón J. A. López-Mora C.. (2024). Sporting mind: the interplay of physical activity and psychological health. Sports 12:37. doi: 10.3390/sports12010037, PMID: 38275986 PMC10819297

[ref27] Muller-Junior I. L. Capraro A. M. (2024). Profile of scientific publications on Muay Thai: an analysis based on Scopus and web of science databases (1998–2021). J. Martial Arts Anthrop. 24, 32–43. doi: 10.14589/ido.24.3.4

[ref28] Nilsen F. A. Bang H. Boe O. Martinsen Ø. L. Lang-Ree O. C. Røysamb E. (2020). The multidimensional self-control scale (MSCS): development and validation. Psychol. Assess. 32, 1057–1074. doi: 10.1037/pas0000950, PMID: 32915001

[ref29] Ong T. F. Ruzmin W. I. (2015); Participation motivation in Muay Thai among Malaysians. In Proceedings of the 2nd international colloquium on sports science, exercise, engineering and technology 2015 (ICoSSEET 2015) Singapore: Springer.

[ref30] Pedrini L. Jennings G. (2021). Cultivating health in martial arts and combat sports pedagogies: a theoretical framework on the care of the self. Front. Sociol. 6:601058. doi: 10.3389/fsoc.2021.601058, PMID: 33869548 PMC8022782

[ref31] Pessina J. E. (2017). Gênero no muay thai: uma luta dentro das artes marciais. Trabalho de conclusão de curso (Licenciatura em Educação Física). Rio Claro: Universidade Estadual Paulista, Instituto de Biociências de Rio Claro.

[ref32] Prasetyo Y. T. Cahigas M. M. L. Patrick E. Rodney M. Nadlifatin R. Persada S. F. (2024). Indonesian martial artists’ preferences in martial arts schools: sustaining business competitiveness through conjoint analysis. PLoS One 19:e0301229. doi: 10.1371/journal.pone.0301229, PMID: 38578778 PMC10997075

[ref33] Quintero A. M. Fuentes-Garcia J. P. Poblete-Valderrama F. de Andrade G. A. P. De la Rosa A. (2024). Body composition, power muscle, and baropodometric assessment in elite Muay Thai athletes. Ido Mov. Cult. 24, 12–22. doi: 10.14589/ido.24.3.2

[ref34] Rapkiewicz J. A. Nunes J. P. Mayhew J. L. Ribeiro A. S. Nabuco H. C. Fávero M. T.. (2018). Effects of Muay Thai training frequency on body composition and physical fitness in healthy untrained women. J. Sports Med. Phys. Fitness 58, 1808–1814. doi: 10.23736/S0022-4707.17.07969-5, PMID: 29111629

[ref35] Saraiva B. T. C. Franchini E. Vanderlei L. C. M. Milanez V. F. Tebar W. R. Beretta V. S.. (2024b). Effects of 16-week Muay Thai practice on cardiovascular parameters in children and adolescents with overweight/obesity. Sport Sci. Health 20, 647–657. doi: 10.1007/s11332-023-01158-5

[ref36] Saraiva B. T. C. Henn R. R. Tebar W. R. dos Santos A. B. Antunes E. P. Ferrari G.. (2024a). Effects of Muay Thai practice on self-esteem, body perception, and quality of life in adolescents with overweight/obesity. Transl. J. Am. Coll. Sports Med. 9:e000269. doi: 10.1249/TJX.0000000000000269

[ref37] Saraiva B. T. C. Prado W. L. D. Vanderlei L. C. M. Milanez V. F. Damato T. M. D. M. Santos A. B. D.. (2022). Acute effects of Muay Thai on blood pressure and heart rate in adolescents with overweight/obesity. Obesities 2, 94–102. doi: 10.3390/obesities2010009

[ref38] Saraiva B. T. C. Ritti-Dias R. M. Farah B. Q. Suetake V. Y. B. Diniz T. A. Costa P.. (2018). Cardiovascular effects of 16 weeks of martial arts training in adolescents. Rev. Bras. Med. Esporte 24, 212–215. doi: 10.1590/1517-869220182403179093

[ref39] Saraiva B. T. C. Ritti-Dias R. M. Scarabottolo C. C. da Silva A. L. F. Tebar W. R. Christofaro D. G. D. (2023). Effects of nine months practice of martial arts on aerobic fitness in children and adolescentes. Sci. Sports 38, 394–400. doi: 10.1016/j.scispo.2022.02.007

[ref40] Schneider M. (2022). An American Buddhist and the healing discipline of Nak Muay: An autoethnographic and critical review of Muay Thai practice and the psychology of self-healing and identity development through the medium of martial arts. Chicago: The Chicago School of Professional Psychology.

[ref41] Soontayatron S. (2025). Exploring serious leisure: the journey of foreigners pursuing Muay Thai training in Bangkok and their socio-cultural adaptation. Ann. Leis. Res. 27, 1–19. doi: 10.1080/11745398.2025.2460801

[ref42] Souza F. B. Farje L. F. (2019). Elementos Fundamentais para um Treinamento Eficaz em Atletas de Muay Thai. J. Cient. Tecnol.

[ref43] Soylu C. Kütük B. (2021). SF-12 Yaşam Kalitesi Ölçeği’nin Türkçe formunun güvenirlik ve geçerlik çalışması. Turk Psikiyatri Derg. 16, 1–9. doi: 10.26466/opus.805108, PMID: 35730511

[ref44] Stepanyan A. Nadoyan A. (2024). Characteristics of the manifestation and exhibition of muay tay athlete’s stress resistance and self-control behavior. Modern Psychol. 7, 3–14. doi: 10.46991/SBMP/2024.7.1.003, PMID: 40191357

[ref46] Turner A. N. (2009). Strength and conditioning for Muay Thai athletes. Strength Cond. J. 31, 78–92. doi: 10.1519/SSC.0b013e3181b99603

[ref47] Utesch T. Dreiskämper D. Naul R. Geukes K. (2018). Understanding physical (in-) activity, overweight, and obesity in childhood: effects of congruence between physical self-concept and motor competence. Sci. Rep. 8:5908. doi: 10.1038/s41598-018-24139-y, PMID: 29651046 PMC5897370

[ref48] Vail P. (2014). Muay Thai: inventing tradition for a national symbol. Sojourn 29, 509–553. doi: 10.1355/sj29.3a

[ref49] Wang S. Lu M. Dong X. Xu Y. (2025b). Does physical activity-based intervention decrease repetitive negative thinking? A systematic review. PLoS One 20:e0319806. doi: 10.1371/journal.pone.0319806, PMID: 40168446 PMC11960971

[ref50] Wang C. T. Tien C. W. Huang W. C. (2025a). Bajiquan martial arts training as physical activity for enhancing physical fitness, body composition, and perceived exercise benefits Bajiquan martial arts training as physical activity for enhancing physical fitness, body composition, and perceived exercise benefit: a quasi-experimental studys. Front. Sports Act. Living 7:1545481. doi: 10.3389/fspor.2025.1545481, PMID: 40230376 PMC11994594

[ref51] Ware J. E. Kosinski M. Keller S. D. (1995). SF-12: How to score the SF-12 physical and mental health summary scales. Boston: The Health Institute, New England Medical Center.

[ref52] Yıldırım M. Özaslan A. (2022). Love of life scale: psychometric analysis of a Turkish adaptation and exploration of its relationship with well-being and personality. Gazi Med. J. 33, 158–162. doi: 10.12996/gmj.2022.36

